# No sex differences in the incidence, risk factors and clinical impact of acute kidney injury in critically ill patients with sepsis

**DOI:** 10.3389/fimmu.2022.895018

**Published:** 2022-07-14

**Authors:** Junnan Peng, Rui Tang, Qian Yu, Daoxin Wang, Di Qi

**Affiliations:** Department of Respiratory and Critical Care Medicine, Second Affiliated Hospital of Chongqing Medical University, Chongqing, China

**Keywords:** sepsis, AKI, sex, intensive care, critically ill

## Abstract

**Background:**

Sex-stratified medicine is an important aspect of precision medicine. We aimed to compare the incidence and risk factors of acute kidney injury (AKI) for critically ill men and women with sepsis. Furthermore, the short-term mortality was compared between men and women with sepsis associated acute kidney injury (SA-AKI).

**Method:**

This was a retrospective study based on the Medical Information Mart for Intensive Care IV database. We used the multivariable logistic regression analysis to evaluate the independent effect of sex on the incidence of SA-AKI. We further applied three machine learning methods (decision tree, random forest and extreme gradient boosting) to screen for the risk factors associated with SA-AKI in the total, men and women groups. We finally compared the intensive care unit (ICU) and hospital mortality between men and women with SA-AKI using propensity score matching.

**Results:**

A total of 6463 patients were included in our study, including 3673 men and 2790 women. The incidence of SA-AKI was 83.8% for men and 82.1% for women. After adjustment for confounders, no significant association was observed between sex and the incidence of SA-AKI (odds ratio (OR), 1.137; 95% confidence interval (CI), 0.949-1.361; *p*=0.163). The machine learning results revealed that body mass index, Oxford Acute Severity of Illness Score, diuretic, Acute Physiology Score III and age were the most important risk factors of SA-AKI, irrespective of sex. After propensity score matching, men had similar ICU and hospital mortality to women.

**Conclusions:**

The incidence and associated risk factors of SA-AKI are similar between men and women, and men and women with SA-AKI experience comparable rates of ICU and hospital mortality. Therefore, sex-related effects may play a minor role in developing SA-AKI. Our study helps to contribute to the knowledge gap between sex and SA-AKI.

## Introduction

Sepsis is one of the most common critical diseases treated in the intensive care unit (ICU) (1). In the United States, the annual incidence of sepsis was 82.7 to 240.4 per 100,000 population (2), while the estimated worldwide incidence was up to 437 per 100,000 population years (3). Sepsis frequently leads to multiorgan dysfunction, and kidney involvement is usual (4). More than half of patients with sepsis develop acute kidney injury (AKI) during hospitalization, and this condition adversely affects patient outcomes (5). Despite years of research, sepsis associated acute kidney injury (SA-AKI) remains an important concern and clinical burden, and the identification of risk factors for SA-AKI is still essential so that targeted strategies may be implemented (6).

Sex-stratified medicine is an important aspect of precision medicine (7). The impact of patient sex on clinical outcomes is an area of intense interest. A large retrospective study of 261,255 critically ill patients revealed that women less than 50 years of age had lower adjusted mortality than men (8). Moreover, different disease processes and outcomes between men and women were also found in some specific disease groups, like coronary artery disease (9), acute ischemic stroke (10) and coronavirus disease 2019 (11). This may be associated with the influence of sex hormones on the modulation of inflammation during immune responses (12). Sexual dimorphism exists in immune processes, leading to the differences in immunomodulation of the cytokine network during inflammatory responses (13, 14). It has been found that excessive inflammation and immune suppression are involved in developing SA-AKI (6, 15). Therefore, the role of sex in SA-AKI should be drawn further attention. Until present, relevant studies are mainly about the effect of sex on mortality of SA-AKI patients, and data on the association between sex and the incidence as well as risk factors of SA-AKI are very scarce.

The primary goal of our study was to assess sex-specific effects on the incidence of AKI in critical patients with sepsis. We further investigated if men and women had differential risk factors associated with SA-AKI, which indicates the non-homogenous management strategies for them. We also aimed to compare the short-term clinical outcomes between men and women with SA-AKI. Our hypothesis was that sex would affect the incidence of SA-AKI, and the risk factors as well as clinical impact of SA-AKI were not similar between men and women.

## Method

### Study design

This study is reported following the REporting of studies Conducted using Observational Routinely collected health Data (RECORD) statement (16). Medical ethical approval and the informed consent were exempted due to the retrospective study design and anonymous information collection.

We extracted patient data from the Medical Information Mart for Intensive Care IV (MIMIC-IV) database (https://mimic.physionet.org/about/mimic/). The description of MIMIC-IV database is available elsewhere (17). In brief, the MIMIC-IV database is a large and publicly available database comprising more than 70,000 patients in the ICU of the Beth Israel Deaconess Medical Center in Boston, MA, USA, between 2008 and 2019. All data were collected before the coronavirus disease 2019 outbreak. To apply for access to the database, we have passed the National Institutes of Health Web-based training course and Protecting Human Research Participants examination (No. 9555299). Data extraction was performed in the Structured Query Language with Navicat Premium (version 15).

### Selection of patients

In the present study, patients older than 18 years of age admitted to the ICU with an ICU length of stay longer than 72 hours were screened for possible inclusion. Since one patient may be admitted to the ICU multiple times, we only counted the first ICU admission due to non-independence of the outcome among subsequent ICU admissions. Patients were included if they met the Sepsis-3.0 definition upon ICU admission. The details of sepsis-3.0 definition include the presence of an infection with signs of organ dysfunction, which are represented by an increase in the Sequential [Sepsis-related] Organ Failure Assessment (SOFA) score of two points or more (18). Infection was identified from the international classification of diseases (ICD)-9 or ICD-10 code in the MIMIC-IV database. We excluded patients if they developed AKI before ICU admission or after 7 days of ICU admission.

### Outcomes

We defined the occurrence of AKI within the first 7 days after ICU admission as the primary outcome of interest. Included patients for whom AKI occurred within 7 days after ICU admission were classified as the AKI group, and the rest of the patients comprised the non-AKI group. The AKI was diagnosed according to the Kidney Disease: Improving Global Outcomes (KDIGO) criteria. KDIGO criteria are as follows (19): increase in serum creatinine (SCr) to ≥ 1.5 times baseline must have occurred within the prior 7 days, or an increase of SCR ≥ 0.3 mg/dl within 48 hours, or urine volume < 0.5 ml/kg/hour for 6 h or more. We used the admission SCr as a baseline value, in accordance with previous studies (20, 21). Patients were categorized into the AKI group (KDIGO stage 3) if they received continuous renal replacement therapy (CRRT) within the first 7 days after ICU admission. Severe AKI was defined as KDIGO stage 2 or higher, otherwise it was defined as mild AKI (22, 23). Secondary outcomes included the ICU and hospital mortality, which were defined as the occurrence of death during the ICU and hospital stay, respectively.

### Sensitivity and subgroup analyses

We did several sensitivity and subgroup analyses for the primary outcomes. First, given the considerable impact of age on sex hormones (7), we conducted a stratified analysis according to age in decades (≤40, (40–50], (50–60], (60–70], (70–80], (80–90] and>90 years). Second, to determine whether the illness severity of AKI would influence the results, we further compared patients without AKI to those with mild AKI or with severe AKI. Lastly, we also performed analyses adjusted for the presence of comorbidities.

### Data extraction

Baseline characteristics within the first 24 h after ICU admission were collected, including age, sex, body mass index (BMI), ethnicity and admission type. Comorbidities including hypertension, coronary atherosclerosis, heart failure, diabetes mellitus, chronic obstructive pulmonary disease (COPD), cerebral infarction, chronic liver disease, chronic kidney disease, and tumors were also collected for analysis based on the recorded ICD-9 or ICD-10 codes in the MIMIC-IV database. The severity as measured by the Sequential Organ Failure Assessment (SOFA) score, the Acute Physiology Score III (APS III) and the Oxford Acute Severity of Illness Score (OASIS) were calculated upon ICU admission. The use of vasopressor, mechanical ventilation, diuretic, aminoglycoside, statin and angiotensin-converting enzyme inhibitors/angiotensin receptor blockers were also recorded. For the AKI group, an intervention was considered positive if conducted before the onset of AKI and negative otherwise; for the non-AKI group, it was based on the records within 7 days of ICU admission. Vital signs included the heart rate, mean arterial pressure, respiratory rate, temperature and peripheral blood oxygen saturation (SpO_2_). Laboratory findings including white blood cell, hemoglobin, platelet, pondus hydrogenii, bicarbonate, blood urea nitrogen, creatinine, potassium, sodium, chloride, glucose, prothrombin time, activated partial thromboplastin time and lactate were measured during the first 24 h in the ICU. If a variable was recorded more than once, we only used the first value for analysis. Detailed information on missing data is available in **Supplementary Table S1**. The random Forest-based imputation method was used to impute missing values for these variables (24). The random forest algorithm was implemented using the R package “missForest” (version 1.4; https://CRAN.R-project.org/package=missForest).

### Statistical analysis

We performed the statistical analysis and created pictures by using R statistical software (version 3.6.1, https://cran.r-project.org/), GraphPad Prizm (version 8.0, San Diego, CA, https://www.graphpad.com/scientific-software/prism/) or SPSS software (version 26.0, IBM, USA, https://www.ibm.com/analytics/spss-statistics-software). For continuous variables, we first investigated the normality using the Kolmogorov-Smirnov test. Normally distributed variables are expressed as mean ± standard deviation (SD) and were compared using Student’s t-test. Non-normally distributed variables are presented as the median and interquartile range (IQR) and were analyzed using the non-parametric Mann–Whitney U test. Categorical variables are expressed as count with percentage (%) and were compared with the chi-square test or Fisher’s exact test as appropriate. All tests were two-tailed. A *p*-value <0.05 was considered significant.

We then used the multivariable logistic regression analyses to assess the independent association between sex and the incidence of SA-AKI. Five different models were built. The first one only included sex, and the second model included sex, age, BMI, ethnicity and admission type. In the third model, we further included comorbidities as covariates. The fourth model additionally included other exposure variables with *p*-value <0.1 in univariate analyses for multivariable analysis, due to their potential influence on the patient’s primary outcome. In the final model, all baseline variables were included in order to provide a comprehensive assessment. In all models, the effect of sex (women=referent) on primary outcome was presented as an odds ratio (OR) with a 95% confidence interval (95%CI).

Further, we applied three machine learning models to screen for the risk factors: decision tree model, random forest model and Extreme Gradient Boosting (XGBoost) model. A decision tree algorithm is a basic classification method that constructs a model based on the feature of data using a tree structure (25). Feature selection, tree generation, and pruning are the basic steps of building decision trees, and an object-relational mapping relationship was eventually generated (26). In this study, we used the Classification and Regression Tree method with the rpart package (version 4.1.16, https://CRAN.R-project.org/package=rpart) for constructing the decision tree in the R language. Random forest is an ensemble algorithm that combines multiple decision trees, and there is no correlation between each decision tree (27). The voting method is used to discriminate and classify data, and the maximum number of votes is taken as the final classification result (28). In this study, we used the RandomForest package (version 4.7-1, https://CRAN.R-project.org/package=randomForest) in the R language for analysis. XGBoost is an ensemble algorithm composed of multiple decision trees and a gradient boost machine (29). The main advantage of XGBoost is to combine multithreading, data compression, and fragmentation methods to improve the efficiency of the algorithm as much as possible (30). In this study, XGBoost was implemented using the xgboost package (version 1.5.2.1, https://CRAN.R-project.org/package=xgboost) in the R language. Machine learning models have exhibited several advantages over conventional statistical methods, and are gradually used in recent studies (31, 32).

Lastly, we estimated the effect of sex on ICU and hospital mortality for patients with SA-AKI. We used propensity score matching (PSM) to balance baseline variables between men and women. Specifically, matching was performed using R package ‘matching’ (version 4.9-11, https://CRAN.R-project.org/package=Matching), with a ratio of 1:1 and a caliper width of 0.1 without replacement. We considered a standardized mean difference (SMD) of less than 0.1 as acceptable. After propensity matching, differences in ICU and hospital mortality were compared between the two groups. This method has been widely used to compare the mortality between different groups, due to its excellent ability to control measured confounding in observational studies (33, 34).

## Results

### Baseline characteristics

**Figure 1:**
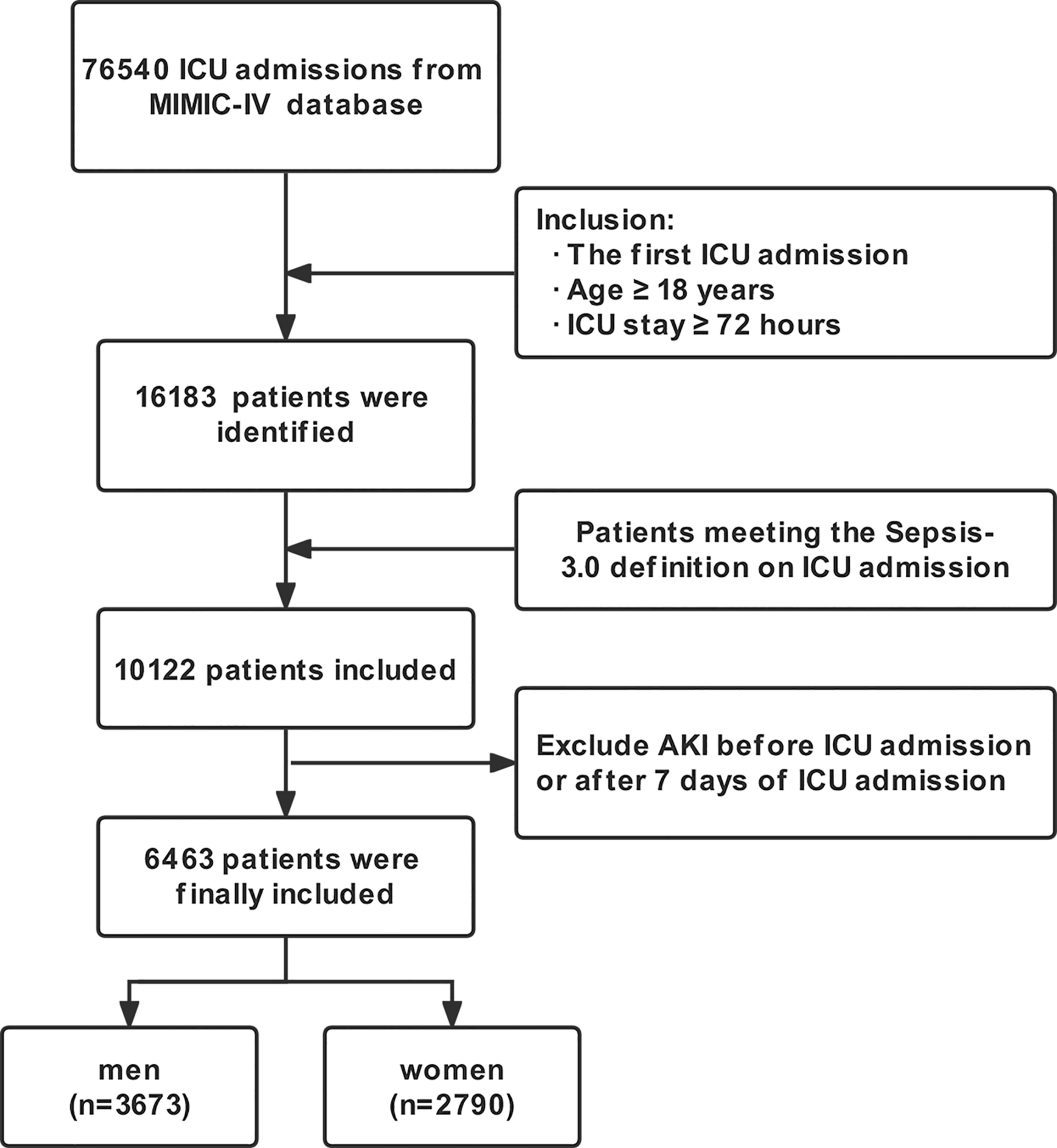
Flow chart of this study. MIMIC-IV, Medical Information Mart for Intensive Care IV database; ICU, Intensive care unit; AKI, Acute kidney injury.

The process of patient selection is shown in **Figure 1**. Finally, a total of 6463 patients were included in our study. Of them, 3673 (56.8%) were men and 2790 (43.2%) were women, with mean age of 66.42 ± 16.58 years. The most common comorbidity was hypertension, followed by heart failure, diabetes mellitus and chronic kidney disease. Men were younger, less likely to have hypertension, heart failure, COPD and cerebral infarction than women; however, men were more likely to have coronary atherosclerosis and chronic kidney disease. Men had lower OASIS and higher SOFA scores than women, but the APS III score was similar between them. In terms of the interventions, more men had mechanical ventilation and statin, while fewer men had diuretic use. The overall incidence of SA-AKI was 83.1% (5368/6463), and men had a slightly higher but not significant incidence than women (83.8% *vs.* 82.1%, *p* = 0.068). The detailed information about baseline characteristics of the total cohort, men and women are shown in **Table 1**.

**Table 1:** Baseline characteristics of patients.

Variables	Total (n=6463)	Men (n=3673)	Women (n=2790)	*p*-value
Age (years)	66.42 ± 16.58	64.86 ± 16.38	68.48 ± 16.61	<0.001	
BMI (kg/m^2^)	29.65 ± 7.50	29.52 ± 7.26	29.82 ± 7.80	0.113	
Ethnicity, n (%)				0.119	
White	4217 (65.2)	2367 (64.4)	1850 (66.3)		
Non-white	2246 (34.8)	1306 (35.6)	940 (33.7)		
Admission type, n (%)				0.715	
Emergency	4003 (61.9)	2282 (62.1)	1721 (61.7)		
Non-emergency	2460 (38.1)	1391 (37.9)	1069 (38.3)		
Comorbidities, n (%)					
Hypertension	2671 (41.3)	1477 (40.2)	1194 (42.8)	0.037	
Coronary atherosclerosis	1098 (17.1)	687 (18.7)	411 (14.7)	<0.001	
Heart failure	2048 (31.7)	1124 (30.6)	924 (33.1)	0.031	
Diabetes mellitus	1708 (26.4)	977 (27.1)	711 (25.5)	0.134	
COPD	561 (8.7)	290 (7.9)	271 (9.7)	0.01	
Cerebral infarction	673 (10.4)	353 (9.6)	320 (11.5)	0.015	
Chronic liver disease	98 (1.5)	61 (1.7)	37 (1.3)	0.276	
Chronic kidney disease	1412 (21.8)	858 (23.4)	554 (19.9)	0.001	
Tumor	1109 (17.2)	619 (16.9)	490 (17.6)	0.453	
Severity scale (at admission)				
APS III	64.89 ± 27.57	64.59 ± 27.91	65.30 ± 27.12	0.307	
OASIS	39.24 ± 9.19	38.77 ± 9.14	39.86 ± 9.21	<0.001	
SOFA	3 (2–5)	3 (2-5)	3 (2-4)	<0.001	
Interventions, n (%)					
Vasopressor use	473 (7.3)	278 (7.6)	195 (7.0)	0.376	
Mechanical ventilation	4087 (63.2)	2391 (65.1)	1696 (60.8)	<0.001	
Diuretic	1616 (25.0)	837 (22.8)	779 (27.9)	<0.001	
Aminoglycoside	135 (2.1)	79 (2.2)	56 (2.0)	0.689	
Statin	1836 (28.4)	1084 (29.5)	752 (27.0)	0.024	
ACEI/ARBs	662 (10.2)	367 (10.0)	295 (10.6)	0.445	
Vital signs					
Heart rate (beats/min)	92.45 ± 21.17	91.99 ± 21.31	93.05 ± 20.97	0.047	
MAP (mmHg)	82.50 ± 19.57	82.80 ± 18.93	82.10 ± 20.38	0.155	
RR (times/min)	20.28 ± 6.32	20.12 ± 6.26	20.50 ± 6.39	0.018	
Temperature (°C)	36.73 ± 1.00	36.76 ± 1.01	36.69 ± 1.00	0.005	
SpO2 (%)	98 (95-100)	98 (95-100)	98 (95-100)	0.927	
Laboratory findings					
WBC (k/uL)	12.3 (8.6-17.2)	12.3 (8.6-17.2)	12.2 (8.5-17.2)	0.692	
Hemoglobin (g/L)	10.60 ± 2.30	10.92 ± 2.41	10.18 ± 2.07	<0.001	
Platelet (k/uL)	202.50 ± 114.86	194.30 ± 111.88	213.30 ± 117.81	<0.001	
PH	7.35 ± 0.10	7.35 ± 0.10	7.35 ± 0.11	0.022	
Bicarbonate (mEq/L)	22.21 ± 5.14	22.23 ± 4.84	22.18 ± 5.51	0.662	
BUN (mg/dL)	22 (15-36)	22 (15-37)	21 (14-34)	<0.001	
Creatinine (mg/dL)	1.1 (0.8-1.7)	1.2 (0.9-1.8)	0.9 (0.7-1.5)	<0.001	
Potassium (mEq/L)	4.24 ± 0.80	4.33 ± 0.81	4.12 ± 0.77	<0.001	
Sodium (mEq/L)	138.79 ± 5.85	138.71 ± 5.73	138.89 ± 6.01	0.213	
Chloride (mEq/L)	104.64 ± 7.18	104.48 ± 7.05	104.83 ± 7.33	0.056	
Glucose (mg/dL)	155.44 ± 82.87	155.75 ± 82.84	155.01 ± 82.93	0.721	
PT (s)	17.20 ± 9.94	17.21 ± 9.53	17.18 ± 10.45	0.918	
APTT (s)	38.97 ± 23.67	39.08 ± 23.56	38.83 ± 23.83	0.672	
Lactate (mmol/L)	1.9 (1.3-2.9)	1.9 (1.3-2.9)	1.8 (1.3-2.8)	0.004	
AKI	5368 (83.1)	3078 (83.8)	2290 (82.1)	0.068	

Data were presented as mean ± standard deviation or median (interquartile range) or numbers (percentages).

ACEI/ARBs, Angiotensin-converting enzyme inhibitors/angiotensin receptor blockers; AKI, Acute kidney injury; APS III, Acute Physiology Score III; APTT, Activated partial thromboplastin time; BMI, Body mass index; BUN, Blood urea nitrogen; COPD, Chronic obstructive pulmonary disease; OASIS, Oxford Acute Severity of Illness Score; PT, Prothrombin time; SOFA, Sequential Organ Failure Assessment; RR, Respiratory rate; WBC, White blood cell.

### Comparison between aki and non-aki groups

In order to search for factors that might influence the incidence of SA-AKI, we compared the baseline characteristics between patients with and without SA-AKI. As shown in **Table 2**, we found that the AKI group was older, had a higher BMI, and was more likely to be admitted from the emergency department. The proportion of patients with heart failure, diabetes mellitus, COPD, chronic liver disease and chronic kidney disease were higher in the AKI group, while more patients had tumors in the non-AKI group. In addition, patients with SA-AKI had higher severity scores on admission when compared to those without SA-AKI, including APS III, OASIS and SOFA scores. Patients with SA-AKI were more likely to have vasopressor use and mechanical ventilation when compared to those without SA-AKI; however, a lower proportion of patients in the AKI group had diuretic, aminoglycoside, statin and ACEI/ARBs. Patients with SA-AKI had significantly higher ICU and hospital mortality rates, compared with the patients without SA-AKI (ICU mortality, 19.84% *vs.* 3.65, *p*<0.001; hospital mortality, 25.09% *vs.* 7.76%, *p*<0.001).

**Table 2:** Comparison between AKI group and non-AKI group.

Variables	non-AKI group (n=1095)	AKI group (n=5368)	*p*-value
Age (years)	61.01 ± 18.98	67.52 ± 15.82	<0.001
Sex (males, %)	595 (54.3)	3078 (57.3)	0.068
BMI (kg/m2)	25.71 ± 4.15	30.46 ± 7.77	<0.001
Ethnicity, n (%)			0.056
White	687 (62.7)	3530 (65.8)	
Non-white	408 (37.3)	1838 (34.2)	
Admission type, n (%)			<0.001
Emergency	356 (32.5)	2104 (39.2)	
Non-emergency	739 (67.5)	3264 (60.8)	
Comorbidities, n (%)			
Hypertension	423 (38.6)	2248 (41.9)	0.047
Coronary atherosclerosis	119 (10.9)	979 (18.2)	<0.001
Heart failure	177 (16.2)	1871 (34.9)	<0.001
Diabetes mellitus	197 (18.0)	1511 (28.1)	<0.001
COPD	67 (6.1)	494 (9.2)	0.001
Cerebral infarction	99 (9.0)	574 (10.7)	0.103
Chronic liver disease	8 (0.7)	90 (1.7)	0.02
Chronic kidney disease	116 (10.6)	1296 (24.1)	<0.001
Tumor	222 (20.3)	887 (16.5)	0.003
Severity scale (at admission)			
APS III	47.98 ± 19.32	68.34 ± 27.73	<0.001
OASIS	32.84 ± 7.72	40.55 ± 8.91	<0.001
SOFA	3 (2-4)	3 (2-5)	<0.001
Interventions, n (%)			
Vasopressor use	51 (4.7)	422 (7.9)	<0.001
Mechanical ventilation use	560 (51.1)	3527 (65.7)	<0.001
Diuretic	506 (46.2)	1110 (20.7)	<0.001
Aminoglycoside	58 (5.3)	77 (1.4)	<0.001
Statin	340 (31.1)	1496 (27.9)	0.033
ACEI/ARBs	253 (23.1)	409 (7.6)	<0.001
Vital signs			
Heart rate (beats/min)	92.65 ± 20.74	92.41 ± 21.25	0.736
MAP (mmHg)	81.15 ± 18.01	82.16 ± 19.86	0.001
RR (times/min)	20.08 ± 5.88	20.32 ± 6.41	0.219
Temperature (°C)	36.88 ± 0.90	36.70 ± 1.02	<0.001
SpO2 (%)	98 (96-100)	98 (95-100)	0.311
Laboratory findings			
WBC (k/uL)	11.4 (7.8-15.8)	12.5 (8.8-17.5)	<0.001
Hemoglobin (g/L)	10.64 ± 2.13	10.59 ± 2.33	0.529
Platelet (k/uL)	208.86 ± 112.72	201.21 ± 115.26	0.045
PH	7.38 ± 0.81	7.35 ± 0.11	<0.001
Bicarbonate (mEq/L)	22.70 ± 4.82	22.11 ± 5.20	<0.001
BUN (mg/dL)	16 (11-27)	23 (15-37)	<0.001
Creatinine (mg/dL)	0.8 (0.6-1.1)	1.1 (0.8-1.8)	<0.001
Potassium (mEq/L)	4.02 ± 0.66	4.28 ± 0.81	<0.001
Sodium (mEq/L)	139.13 ± 5.87	138.72 ± 5.84	0.035
Chloride (mEq/L)	105.11 ± 7.13	104.54 ± 7.18	0.016
Glucose (mg/dL)	141.06 ± 69.98	158.37 ± 84.97	<0.001
PT (s)	15.34 ± 6.55	17.57 ± 10.46	<0.001
APTT (s)	34.59 ± 18.37	39.87 ± 24.52	<0.001
Lactate (mmol/L)	1.7 (1.3-2.3)	1.9 (1.3-3)	<0.001

Data were presented as mean ± standard deviation or median (interquartile range) or numbers (percentages).

ACEI/ARBs, Angiotensin-converting enzyme inhibitors/angiotensin receptor blockers; AKI, Acute kidney injury; APS III, Acute Physiology Score III; APTT, Activated partial thromboplastin time; BMI, Body mass index; BUN, Blood urea nitrogen; COPD, Chronic obstructive pulmonary disease; OASIS, Oxford Acute Severity of Illness Score; PT, Prothrombin time; SOFA, Sequential Organ Failure Assessment; RR, Respiratory rate; WBC, White blood cell.

### Association between sex and SA-AKI

**Figure 2:**
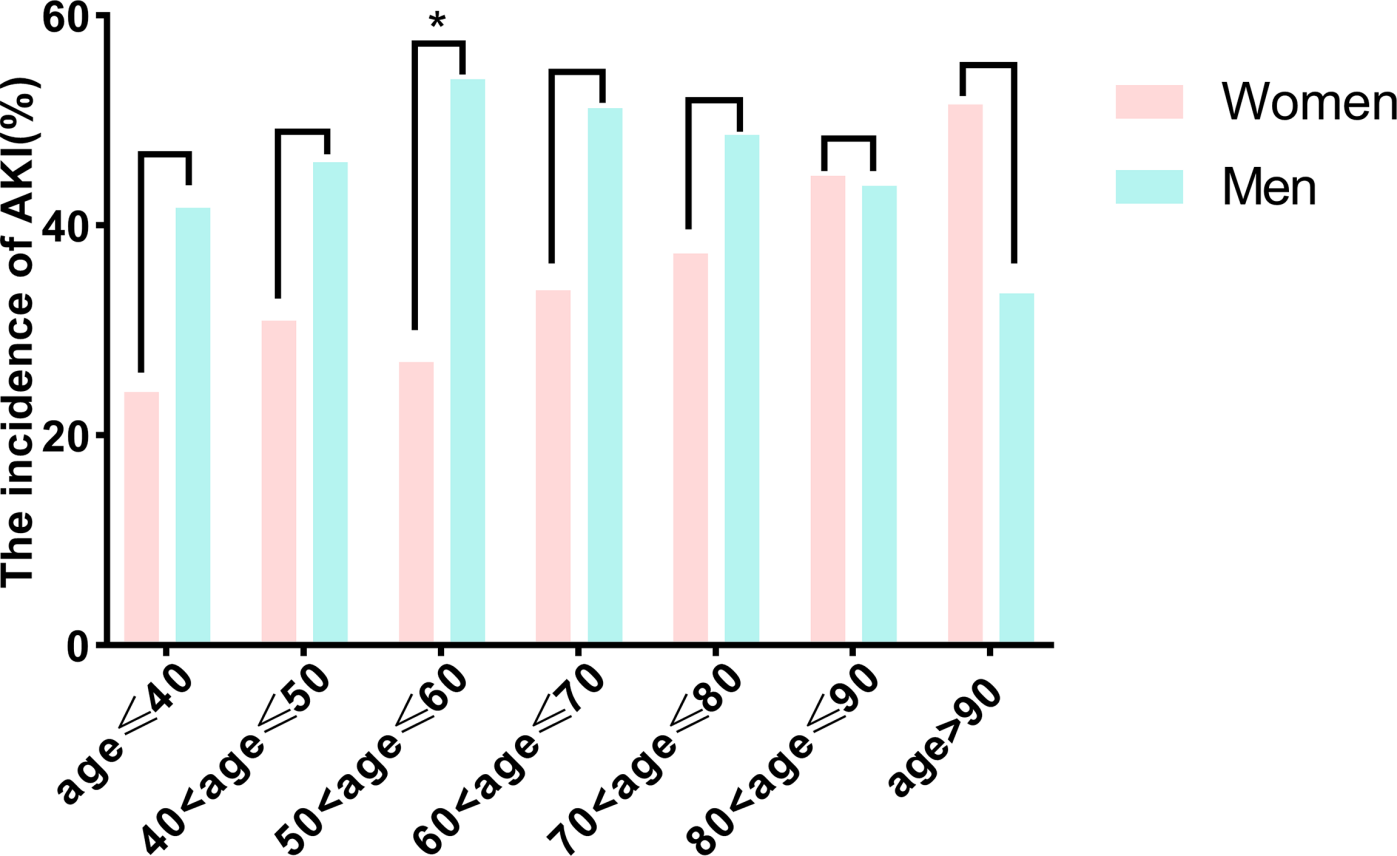
Comparison of the incidence of acute kidney injury between men and women according to age in decades. *p<0.05. AKI, Acute kidney injury.

**Figure 3:**
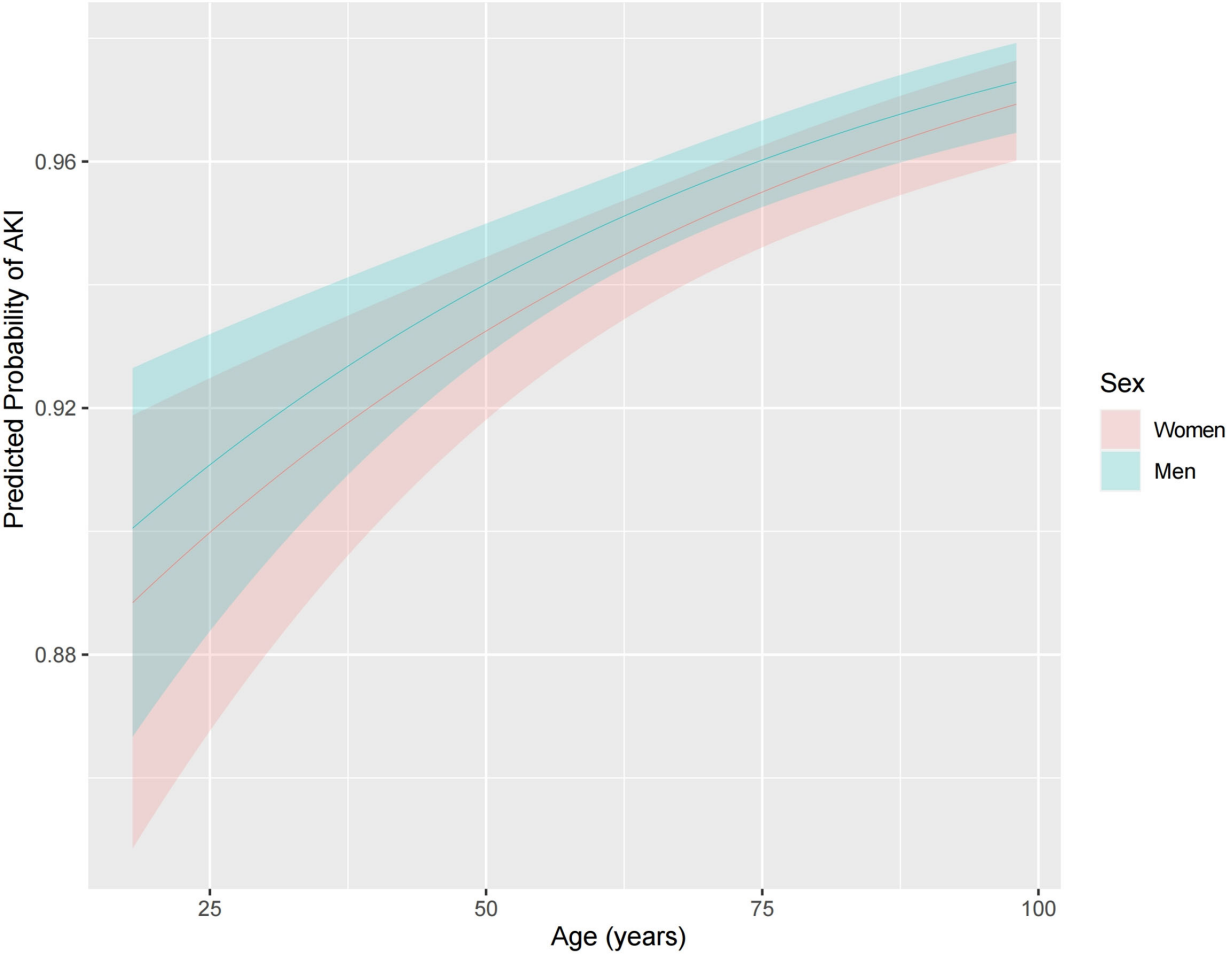
Effect of age on the adjusted incidence of acute kidney injury in men and women. Models were adjusted for all variables, including age, BMI, ethnicity, admission type, hypertension, coronary atherosclerosis, heart failure, diabetes mellitus, COPD, cerebral infarction, chronic liver disease, chronic kidney disease, tumor, APS III, OASIS, SOFA, heart rate, MAP, RR, temperature, SpO2, WBC, hemoglobin, platelet, PH, bicarbonate, BUN, creatinine, potassium, sodium, chloride, glucose, PT, APTT, lactate, vasopressor use, mechanical ventilation use, diuretic, aminoglycoside, statin, ACEI/ARBs. ACEI/ARBs, Angiotensin-converting enzyme inhibitors/angiotensin receptor blockers; AKI, Acute kidney injury; APS III, Acute Physiology Score III; APTT, Activated partial thromboplastin time; BMI, Body mass index; BUN, Blood urea nitrogen; COPD, Chronic obstructive pulmonary disease; OASIS, Oxford Acute Severity of Illness Score; PT, Prothrombin time; SA-AKI, Sepsis associated acute kidney injury; SOFA, Sequential Organ Failure Assessment; RR, Respiratory rate; WBC, White blood cell.

We examined the association between sex and SA-AKI in univariate and extended logistic regression models. We found that men were associated with a higher likelihood of having SA-AKI, but this association became insignificant in the full adjusted model (OR, 1.137; 95% CI, 0.949-1.361; *p* = 0.163; **Table 3**). Similarly, multiple logistic regression revealed that no significant differences in SA-AKI incidence between sexes when comparing patients without SA-AKI to those with mild SA-AKI or with severe SA-AKI (non-AKI *vs.* mild AKI, OR 1.161, 95% CI 0.962-1.402, *p* = 0.120, **Table S2**; non-AKI *vs.* severe AKI, OR 1.175; 95% CI 0.912-1.513; *p* = 0.211, **Table S3**). Then, we examined the effect of age on sex-related outcomes. When age was stratified per decade, there was no association between sexes and SA-AKI incidence except for higher rates for men in those aged 50 to 60 years (men *vs.* women, 19.23% *vs.* 20.66%, *p* = 0.196; **Figure 2**). However, in logistic regression adjusted for all baseline variables, the SA-AKI incidence in men and women was approximately the same for all ages (**Figure 3**). Additionally, we conducted a series of subgroup analyses to determine the effect of comorbidity on the incidence of SA-AKI. The results remained consistent except for patients with cerebral infarction (men *vs.* women, OR 2.062, 95%CI 1.088-3.905, *p*=0.026, **Table S4**).

**Table 3:** Multivariate logistic regression analysis of sex for SA-AKI.

Model	OR (95%CI)	*p-*value
Model 1	1.129 (0.991-1.287)	0.068
Model 2	1.266 (1.100-1.457)	0.001
Model 3	1.230 (1.067-1.419)	0.004
Model 4	1.126 (0.943-1.344)	0.190
Model 5	1.137 (0.949-1.361)	0.163

Adjusted covariates: Model 1= sex (women=referent).

Model 2= Model 1 + age, BMI, ethnicity and admission type.

Model 3=Model 2 + comorbidities (hypertension, coronary atherosclerosis, heart failure, diabetes mellitus, COPD, cerebral infarction, chronic liver disease, chronic kidney disease, tumor).

Model 4=Model 3 + other variables with p<0.1 in the univariate analysis (APS III, OASIS, SOFA, MAP, temperature, WBC, platelet, PH, bicarbonate, BUN, creatinine, potassium, sodium, chloride, glucose, PT, APTT, lactate, vasopressor use, mechanical ventilation use, diuretic, aminoglycoside, statin, ACEI/ARBs).

Model 5 was adjusted for all baseline variables.

ACEI/ARBs, Angiotensin-converting enzyme inhibitors/angiotensin receptor blockers; APS III, Acute Physiology Score III; APTT, Activated partial thromboplastin time; BMI, Body mass index; BUN, Blood urea nitrogen; CI, Confidence interval; COPD, Chronic obstructive pulmonary disease; OASIS, Oxford Acute Severity of Illness Score; OR, Odds ratio; PT, Prothrombin time; SA-AKI, Sepsis associated acute kidney injury; SOFA, Sequential Organ Failure Assessment; RR, Respiratory rate; WBC, White blood cell.

### Risk factors for patients with SA-AKI

**Figure 4:**
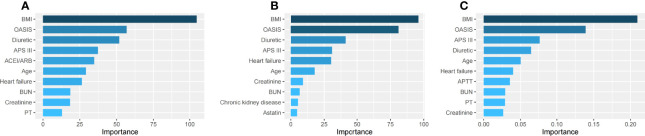
The top ten important factors of SA-AKI for the entire cohort in the decision tree model **(A)**, random forest model **(B)** and extreme gradient boosting model **(C)**. Models were adjusted for all variables, including age, BMI, ethnicity, admission type, hypertension, coronary atherosclerosis, heart failure, diabetes mellitus, COPD, cerebral infarction, chronic liver disease, chronic kidney disease, tumor, APS III, OASIS, SOFA, heart rate, MAP, RR, temperature, SpO2, WBC, hemoglobin, platelet, PH, bicarbonate, BUN, creatinine, potassium, sodium, chloride, glucose, PT, APTT, lactate, vasopressor use, mechanical ventilation use, diuretic, aminoglycoside, statin, ACEI/ARBs. ACEI/ARBs, Angiotensin-converting enzyme inhibitors/angiotensin receptor blockers; AKI, Acute kidney injury; APS III, Acute Physiology Score III; APTT, Activated partial thromboplastin time; BMI, Body mass index; BUN, Blood urea nitrogen; COPD, Chronic obstructive pulmonary disease; OASIS, Oxford Acute Severity of Illness Score; PT, Prothrombin time; SA-AKI, Sepsis associated acute kidney injury; SOFA, Sequential Organ Failure Assessment; RR, Respiratory rate; WBC, White blood cell.

**Figure 5:**
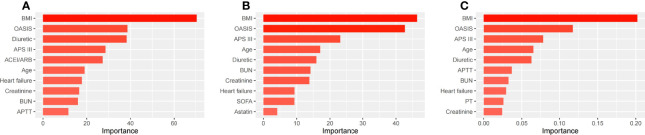
The top ten important factors of SA-AKI for men in the decision tree model **(A)**, random forest model **(B)** and extreme gradient boosting model **(C)**. Models were adjusted for all variables, including age, BMI, ethnicity, admission type, hypertension, coronary atherosclerosis, heart failure, diabetes mellitus, COPD, cerebral infarction, chronic liver disease, chronic kidney disease, tumor, APS III, OASIS, SOFA, heart rate, MAP, RR, temperature, SpO2, WBC, hemoglobin, platelet, PH, bicarbonate, BUN, creatinine, potassium, sodium, chloride, glucose, PT, APTT, lactate, vasopressor use, mechanical ventilation use, diuretic, aminoglycoside, statin, ACEI/ARBs. ACEI/ARBs, Angiotensin-converting enzyme inhibitors/angiotensin receptor blockers; AKI, Acute kidney injury; APS III, Acute Physiology Score III; APTT, Activated partial thromboplastin time; BMI, Body mass index; BUN, Blood urea nitrogen; COPD, Chronic obstructive pulmonary disease; OASIS, Oxford Acute Severity of Illness Score; PT, Prothrombin time; SA-AKI, Sepsis associated acute kidney injury; SOFA, Sequential Organ Failure Assessment; RR, Respiratory rate; WBC, White blood cell.

**Figure 6:**
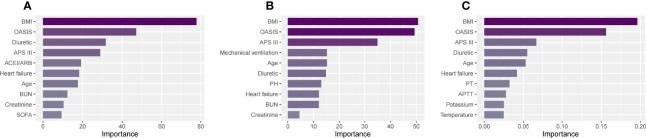
The top ten important factors of SA-AKI for women in the decision tree model **(A)**, random forest model **(B)** and extreme gradient boosting model **(C)**. Models were adjusted for all variables, including age, BMI, ethnicity, admission type, hypertension, coronary atherosclerosis, heart failure, diabetes mellitus, COPD, cerebral infarction, chronic liver disease, chronic kidney disease, tumor, APS III, OASIS, SOFA, heart rate, MAP, RR, temperature, SpO2, WBC, hemoglobin, platelet, PH, bicarbonate, BUN, creatinine, potassium, sodium, chloride, glucose, PT, APTT, lactate, vasopressor use, mechanical ventilation use, diuretic, aminoglycoside, statin, ACEI/ARBs. ACEI/ARBs, Angiotensin-converting enzyme inhibitors/angiotensin receptor blockers; AKI, Acute kidney injury; APS III, Acute Physiology Score III; APTT, Activated partial thromboplastin time; BMI, Body mass index; BUN, Blood urea nitrogen; COPD, Chronic obstructive pulmonary disease; OASIS, Oxford Acute Severity of Illness Score; PT, Prothrombin time; SA-AKI, Sepsis associated acute kidney injury; SOFA, Sequential Organ Failure Assessment; RR, Respiratory rate; WBC, White blood cell.

We used three machine learning methods (decision tree model, random forest model and XGBoost model) to evaluate risk factors of SA-AKI in patients with sepsis. Of all patients, BMI, OASIS, diuretic, APS III and age had the five highest values in importance of the models (**Figures 4A** **–C**). Similar results were also found in men (**Figures 5A** **–C**) and women (**Figures 6A** **–C**).

### Sex-related short-term mortality in patients with SA-AKI

**Figure 7:**
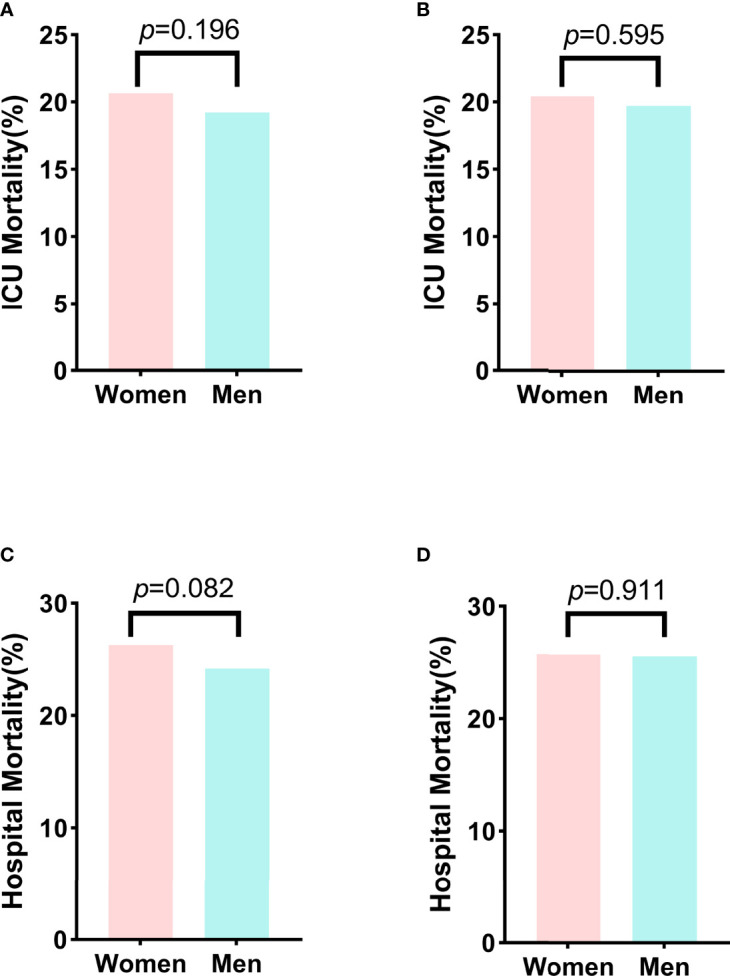
Comparison of the ICU and hospital mortality between men and women before matching and after matching. **(A)** The ICU mortality between men and women before matching. **(B)** The ICU mortality between men and women after matching. **(C)** The hospital mortality between men and women before matching. **(D)** The hospital mortality between men and women after matching. ICU, Intensive care unit.

We finally assessed the effect of sex on in-hospital and ICU mortality in patients with SA-AKI. Before propensity-score matching, age, the proportion of patients with coronary atherosclerosis, OASIS, SOFA, the proportion of mechanical ventilation and diuretic usage, hemoglobin, platelet, BUN, creatinine and potassium were different between the men and women groups (**Table 4**). We found that men had lower but not statistically significant ICU and hospital mortality than women (ICU mortality, men *vs.* women, 19.23% *vs.* 20.66%, *p* = 0.196, **Figure 7A**; hospital mortality, men *vs.* women, 24.20% *vs.* 26.29%, *p* = 0.082, **Figure 7C**). With the use of propensity-score matching (1:1 matching ratio), 1866 pairs of matched SA-AKI patients were created. After matching, we found no significant imbalance between the two groups, with the standardized mean difference being<0.1 for all variables (**Figure S1**; **Table 4**). No differences in ICU and hospital mortality between men and women were found (ICU mortality, men *vs.* women, 19.72% *vs.* 20.42%, *p* = 0.595, **Figure 7B**; hospital mortality, men *vs.* women, 25.62% *vs.* 25.77%, *p* = 0.991, **Figure 7D**).

**Table 4:** Comparisons of baseline SA-AKI patient characteristics before and after propensity score matching.

Variables	Before propensity score matching			After propensity score matching		
	Men (n=3078)	Women (n=2290)	SMD	Men (n=1866)	Women (n=1866)	SMD
Age (years)	65.95 ± 15.70	69.65 ± 15.73	0.235	68.55 ± 15.10	68.66 ± 16.19	0.007
BMI (kg/m^2^)	30.22 ± 7.54	30.77 ± 8.06	0.07	30.44 ± 7.98	30.46 ± 7.50	0.003
Ethnicity, n (%)			0.013			0.009
White	2016 (65.5)	1514 (66.1)		1243 (66.6)	1235 (66.2)	
Non-white	1062 (34.5)	776 (33.9)		623 (33.4)	631 (33.8)	
Admission type, n (%)			0.002			0.007
Emergency	1205 (39.1)	899 (39.3)		732 (39.2)	726 (38.9)	
Non-emergency	1873 (60.9)	1391 (60.7)		1134 (60.8)	1140 (61.1)	
Comorbidities, n (%)						
Hypertension	1261 (41.0)	987 (43.1)	0.043	771 (41.3)	796 (42.7)	0.027
Coronary atherosclerosis	612 (19.9)	367 (16.0)	0.101	300 (16.1)	325 (17.4)	0.036
Heart failure	1034 (33.6)	837 (36.6)	0.062	669 (35.9)	652 (34.9)	0.019
Diabetes mellitus	881 (28.6)	630 (27.5)	0.025	533 (28.6)	534 (28.6)	0.001
COPD	252 (8.2)	242 (10.6)	0.082	179 (9.6)	183 (9.8)	0.007
Cerebral infarction	306 (9.9)	268 (11.7)	0.057	208 (11.1)	207 (11.1)	0.002
Chronic liver disease	56 (1.8)	34 (1.5)	0.026	30 (1.6)	30 (1.6)	<0.001
Chronic kidney disease	790 (25.7)	506 (22.1)	0.084	450 (24.1)	440 (23.6)	0.013
Tumor	493 (16.0)	394 (17.2)	0.032	318 (17.0)	315 (16.9)	0.004
Severity scale (at admission)						
APS III	68.00 ± 28.03	68.80 ± 27.32	0.029	68.81 ± 27.26	68.28 ± 27.51	0.019
OASIS	40.02 ± 8.86	41.26 ± 8.93	0.139	40.84 ± 8.72	40.70 ± 8.91	0.016
SOFA	3 (2-5)	3 (2-5)	0.13	3 (2-5)	3 (2-5)	0.034
Interventions, n (%)						
Vasopressor use	252 (8.2)	170 (7.4)	0.028	136 (7.3)	142 (7.6)	0.012
Mechanical ventilation	2085 (67.7)	1442 (63.0)	0.1	1191 (63.8)	1215 (65.1)	0.027
Diuretic	579 (18.8)	531 (23.2)	0.108	417 (22.3)	410 (22.0)	0.009
Aminoglycoside	49 (1.6)	28 (1.2)	0.031	20 (1.1)	26 (1.4)	0.029
Statin	894 (29.0)	602 (26.3)	0.062	518 (27.8)	514 (27.5)	0.005
ACEI/ARBs	229 (7.4)	180 (7.9)	0.016	136 (7.3)	142 (7.6)	0.012
Vital signs						
Heart rate (beats/min)	91.95 ± 21.40	93.03 ± 21.04	0.051	92.47 ± 21.45	92.28 ± 70.70	0.009
MAP (mmHg)	82.55 ± 19.27	81.64 ± 20.63	0.045	81.82 ± 18.94	81.91 ± 20.52	0.004
RR (times/min)	20.16 ± 6.34	20.54 ± 6.50	0.058	20.37 ± 6.50	20.43 ± 6.47	0.01
Temperature (°C)	36.73 ± 1.03	36.66 ± 1.02	0.072	36.68 ± 1.05	36.68 ± 1.03	0.006
SpO2 (%)	98 (95-100)	98 (95-100)	0.005	99 (95-100)	98 (95-100)	0.028
Laboratory findings						
WBC (k/uL)	12.5 (8.8-17.5)	12.4 (8.7-17.5)	0.007	12.4 (8.7-17.4)	12.4 (8.7-17.4)	0.013
Hemoglobin (g/L)	10.91 ± 2.45	10.16 ± 2.09	0.333	10.32 ± 2.29	10.38 ± 2.08	0.028
Platelet (k/uL)	192.59 ± 111.05	212.79 ± 119.73	0.175	199.50 ± 121.63	202.08 ± 107.13	0.022
PH	7.34 ± 0.11	7.35 ± 0.11	0.037	7.35 ± 0.10	7.35 ± 0.11	0.021
Bicarbonate (mEq/L)	22.10 ± 4.87	22.11 ± 5.61	0.002	22.18 ± 4.85	22.10 ± 5.56	0.016
BUN (mg/dL)	24 (16-39)	22 (14-35)	0.137	24 (16-37)	22 (14-36)	0.087
Creatinine (mg/dL)	1.2 (0.9-1.9)	1 (0.7-1.6)	0.231	1.2 (0.9-1.7)	1 (0.7-1.7)	0.032
Potassium (mEq/L)	4.36 ± 0.83	4.17 ± 0.78	0.24	4.23 ± 0.73	4.21 ± 0.80	0.025
Sodium (mEq/L)	138.65 ± 5.72	138.80 ± 6.00	0.025	138.71 ± 5.91	138.83 ± 6.01	0.02
Chloride (mEq/L)	104.38 ± 7.08	104.75 ± 7.32	0.05	104.59 ± 7.15	104.75 ± 7.34	0.022
Glucose (mg/dL)	159.12 ± 85.96	157.36 ± 83.63	0.021	155.56 ± 82.71	157.99 ± 83.72	0.029
PT (s)	17.56 ± 9.96	17.59 ± 11.1	0.003	17.60 ± 9.74	17.40 ± 10.60	0.02
APTT (s)	40.07 ± 24.48	39.59 ± 24.58	0.02	40.10 ± 24.83	39.69 ± 24.97	0.017
Lactate (mmol/L)	1.9 (1.4-3)	1.9 (1.3-3)	0.054	1.9 (1.3-2.9)	1.9 (1.3-3)	<0.001

Data were presented as mean ± standard deviation or median (interquartile range) or numbers (percentages).

ACEI/ARBs, Angiotensin-converting enzyme inhibitors/angiotensin receptor blockers; AKI, Acute kidney injury; APS III, Acute Physiology Score III; APTT, Activated partial thromboplastin time; BMI, Body mass index; BUN, Blood urea nitrogen; COPD, Chronic obstructive pulmonary disease; OASIS, Oxford Acute Severity of Illness Score; PT, Prothrombin time; SA-AKI, Sepsis associated acute kidney injury; SMD, Standardized mean difference; SOFA, Sequential Organ Failure Assessment; RR, Respiratory rate; WBC, White blood cell.

## Discussion

In this large retrospective study, we compared the incidence of SA-AKI and risk factors associated with SA-AKI for critically ill men and women with sepsis. The sex-specific clinical outcomes were also examined in men versus women with SA-AKI. To our surprise, we found that men and women had similar SA-AKI incidence, and the association between sex and the incidence of SA-AKI was insignificant in the full-adjusted model. The risk factors of SA-AKI in men were almost consistent with those in women. The ICU and hospital mortality were comparable between men and women with SA-AKI. Therefore, the main results were contrary to our original hypothesis. Our study suggested that sex may not act as an essential factor of SA-AKI, and the clinical management of SA-AKI could be the same for men and women.

Knowledge of sex differences is an essential ingredient in developing precision medicine (7). It has been found that sex could affect the manifestation and pathophysiology of many diseases (9–11). The impact of sex on outcomes in patients with sepsis has been widely investigated, yielding conflicting results (35–38). It has been found that women generally have more advantageous immunological responses compared to men by the effect of their sexual hormones (13, 39). Animal studies have demonstrated that testosterone depletion or estrogen supplementation exerts beneficial effects on sepsis (40). Sexual immunomodulation modulates the release of pro-inflammatory and anti-inflammatory cytokines, which is associated with the subsequent multiorgan failure (14, 40). Moreover, ischemia–reperfusion injury is another common source of AKI. Experimental studies suggested that sex hormones regulate cellular pathways involved in kidney ischemia–reperfusion injury and have been implicated in defining AKI susceptibility (41, 42). Kidney is the organ most often involved in sepsis, therefore the effect of sex in SA-AKI should be further explored.

In univariate analysis, we found that the incidence of SA-AKI was lower in women than that in men, but this difference did not reach statistical significance. After adjusting for relevant confounders, we could not determine the significant sex-specific difference in SA-AKI incidence. The results were consistent and stable in patients with different age groups and the degree of disease severity. Moreover, in the analysis of patients with or without different comorbidities, men and women still had similar incidences of SA-AKI except for those with cerebral infarction. One potential explanation for this may include the sample size is relatively small, thus increasing the chance of false‐positive outcomes. Future studies specifically designed to avoid sample bias are needed to validate this result. It is generally assumed that men had a higher incidence of sepsis, but the research about the relationship between sex and sepsis prognosis failed to reach consistent conclusions (35–38). Ponce-Alonso et al. found that men with sepsis had worse clinical characteristics when admitted to the ICU, but sex had no influence on mortality (35). They compared their results with the last 15 years’ relevant published studies, and indicated that geographic location and case definitions were important confounders. Our study was a single-center study with consistent disease definitions, which could minimize the effects of the above factors. A recent study of 17,146 septic patients also reported similar findings (36). The role of sex in kidney disease remains a topic of broad interest. Population-based studies indicated that women have a higher prevalence of chronic kidney disease overall, but men were more likely to experience adverse cardiovascular events and death (43–45). Moreover, men were twice as likely to develop kidney cancer than women and had a higher mortality rate (46). These findings could be partly explained by the difference in sex hormones and the diseases mentioned above are chronic conditions. Additionally, exogenous hormone therapy was associated with the incidence of AKI in patients with prostate cancer (47). Therefore, the sex-related effects may play a minor role in the clinical course of SA-AKI due to the acute situation in patients with sepsis and AKI. Based on our study design, this finding must remain descriptive, and the explanation of the causes remains speculative.

Interestingly, the risk factor analysis showed a strong positive association between BMI and the incidence of SA-AKI. A study of almost 15,000 critically ill patients suggested that each 5 kg/m2 increase in body mass index was associated with a 10% risk of more severe AKI (48). And morbidly obese (defined as BMI >40) has been demonstrated to be an independent risk factor for AKI (49). The following reasons could be attributed (49, 50): First, obesity would increase renal blood flow and induce glomerular hyperfiltration, leading to structural changes in glomerular cells and thus increasing the risk of SA-AKI. Second, higher sympathetic and renin-angiotensin-system activities were found in obese patients, which could enhance kidney damage. Finally, adipose tissue secretes various pro- and anti-inflammatory adipokines that may influence the balance of prostaglandins and thromboxane in the kidney. The biological behavior of the mesangial cells may accordingly change, and thereby partially responsible for the development of SA-AKI.

OASIS and APS III are common scoring systems used to quantify the severity of illness across hospitalized patients (51, 52). Wang et al. have found that OASIS presented good discrimination and calibration in predicting prognosis of AKI (52). Indeed, these scoring systems contain rich clinical information and have been validated in various studies. Our study further supports their predictive value in SA-AKI. Although many studies have explored the effect of diuretic on the development or progression of AKI, no consistent conclusion has been achieved (53). Victor et al. observed that the need for diuretic was positively associated with AKI, and they suggested that this was due to the diuretic use may be a reflection of more severe forms of AKI (e.g., oliguric/anuric AKI) instead of a direct cause of AKI (54). More targeted research is needed in this area for a more definitive account. In addition, previous studies have proven that older age was associated with a significantly higher incidence of AKI (6). There are many reasons for this, but the primary one is the increased vulnerability of the kidney to stressors and insults with increasing age (55).

In the present study, it was not unexpected to observe that SA-AKI was associated with increased ICU and hospital mortality in patients with sepsis, regardless of sex. Therefore, we further compared the short-term mortality between men and women with SA-AKI. Our results revealed that men and women had similar ICU and hospital mortality after propensity score matching. To date, few studies have explored the risk factors of mortality in SA-AKI. Passos et al. found that norepinephrine utilization, liver failure, medical condition, lactate level, and pre-dialysis creatinine level were associated with early mortality in SA-AKI patients treated with CRRT (56). Two other studies also revealed that comorbidities, disease severity and certain drugs are the main risk factors for mortality in SA-AKI (57, 58). Taken together, sex is not a predominant factor affecting the prognosis of patients with SA-AKI.

Some limitations pertain to our study. First, our study was an observational retrospective design that precludes any definitive inference about causality. Second, the care of patients with sepsis may have changed during the study period, which might have affected the incidence of SA-AKI in these patients. Third, we only considered traditional parameters and did not include some sex-specific variables, such as hormone levels, which may help explain the potential mechanism of our results. Fourth, our study only included ICU patients, thus caution should be taken when attempting to generalize our findings to the whole population. These limitations could be overcome by more in-depth, large-scale, and prospective studies in the future.

## Conclusions

In this study, we could not detect the significant sex-specific difference in SA-AKI incidence based on data from critically ill patients with sepsis. BMI, OASIS, diuretic, APS III and age are all the most common risk factors of SA-AKI for the total, men and women groups. The ICU and hospital mortality are comparable between men and women with SA-AKI. Therefore, our study indicates that sex plays a minor role in the clinical course of SA-AKI, and further prospective studies are needed to validate our findings.

## Data availability statement

The raw data supporting the conclusions of this article will be made available by the authors, without undue reservation.

## Ethics statement

Ethical review and approval was not required for the study on human participants in accordance with the local legislation and institutional requirements. Written informed consent for participation was not required for this study in accordance with the national legislation and the institutional requirements.

## Author contributions

Conceptualization and supervision: DW and DQ. Methodology and software: JP and RT. Writing-Original draft preparation: JP, RT and QY. Writing- Reviewing and Editing: all authors. Final approval for publication: all authors

## Funding

This research was funded by National Natural Science Foundation of Chongqing, China(Grant NO. CQYC2020020321).

## Conflict of interest

The authors declare that the research was conducted in the absence of any commercial or financial relationships that could be construed as a potential conflict of interest.

## Publisher’s note

All claims expressed in this article are solely those of the authors and do not necessarily represent those of their affiliated organizations, or those of the publisher, the editors and the reviewers. Any product that may be evaluated in this article, or claim that may be made by its manufacturer, is not guaranteed or endorsed by the publisher.
