# Supplementary material for: No sex differences in the incidence, risk factors and clinical impact of acute kidney injury in critically ill patients with sepsis
Source: Front Immunol. 2022 Jul 14;13:895018. doi: 10.3389/fimmu.2022.895018 (PMC9329949; doi:10.3389/fimmu.2022.895018)

**Supplementary materials**

Table S1. Percentage of missing data in variables of interest

| Variables | Missing value (n=6463) | Percent (%) |
| --- | --- | --- |
| Age (years) | 0 | 0 |
| Sex (males, %) | 0 | 0 |
| BMI (kg/m2) | 1771 | 27.4 |
| Ethnicity | 0 | 0 |
| Admission type | 0 | 0 |
| Hypertension | 0 | 0 |
| Coronary atherosclerosis | 0 | 0 |
| Heart failure | 0 | 0 |
| Diabetes mellitus | 0 | 0 |
| COPD | 0 | 0 |
| Cerebral infarction | 0 | 0 |
| Chronic liver disease | 0 | 0 |
| Chronic kidney disease | 0 | 0 |
| Tumor | 0 | 0 |
| APS III | 0 | 0 |
| OASIS | 0 | 0 |
| SOFA | 0 | 0 |
| Heart rate (beates/min) | 1 | 0.02 |
| MAP (mmHg) | 0 | 0 |
| RR (times/min) | 1 | 0.02 |
| Temperature (℃) | 16 | 0.25 |
| SpO2 (%) | 0 | 0 |
| WBC (k/uL) | 4 | 0.06 |
| Hemoglobin (g/L) | 4 | 0.06 |
| Platelet (k/uL) | 4 | 0.06 |
| PH | 459 | 7.1 |
| Bicarbonate (mEq/L) | 4 | 0.06 |
| BUN (mg/dL) | 4 | 0.06 |
| Creatinine (mg/dL) | 4 | 0.06 |
| Potassium (mEq/L) | 4 | 0.06 |
| Sodium (mEq/L) | 4 | 0.06 |
| Chloride (mEq/L) | 4 | 0.06 |
| Glucose (mg/dL) | 5 | 0.07 |
| PT (s) | 121 | 1.87 |
| APTT (s) | 126 | 1.95 |
| Lactate (mmol/L) | 618 | 9.56 |
| Vasopressor use | 0 | 0 |
| Mechanical ventilation use | 0 | 0 |
| Diuretic | 0 | 0 |
| Aminoglycoside | 0 | 0 |
| Statin | 0 | 0 |
| ACEI/ARBs | 0 | 0 |

**Abbreviations**: ACEI/ARBs, Angiotensin-converting enzyme inhibitors/angiotensin receptor blockers; AKI, Acute kidney injury; APS III, Acute Physiology Score III; APTT, Activated partial thromboplastin time; BMI, Body mass index; BUN, Blood urea nitrogen; COPD, Chronic obstructive pulmonary disease; OASIS, Oxford Acute Severity of Illness Score; PT, Prothrombin time; SOFA, Sequential Organ Failure Assessment; RR, Respiratory rate; WBC, White blood cell.

Table S2. Multivariate logistic regression analysis of sex for mild SA-AKI

| Model | OR (95%CI) | *p-*value |
| --- | --- | --- |
| Model 1 | 1.123(0.980-1.287) | 0.094 |
| Model 2 | 1.290(1.113-1.496) | 0.001 |
| Model 3 | 1.270(1.093-1.476) | 0.002 |
| Model 4 | 1.152(0.957-1.387) | 0.134 |
| Model 5 | 1.161(0.962-1.402) | 0.120 |

**Notes:** Adjusted covariates: Model 1= sex (women=referent).

Model 2= Model 1 + age, BMI, ethnicity and admission type.

Model 3=Model 2 + comorbidities (hypertension, coronary atherosclerosis, heart failure, diabetes mellitus, COPD, cerebral infarction, chronic liver disease, chronic kidney disease, tumor).

Model 4=Model 3 + other variables with *p*<0.1 in the univariate analysis (APS III, OASIS, SOFA, MAP, temperature, WBC, platelet, PH, bicarbonate, BUN, creatinine, potassium, sodium, chloride, glucose, PT, APTT, lactate, vasopressor use, mechanical ventilation use, diuretic, aminoglycoside, statin, ACEI/ARBs).

Model 5 was adjusted for all baseline variables.

**Abbreviations:** ACEI/ARBs, Angiotensin-converting enzyme inhibitors/angiotensin receptor blockers; APS III, Acute Physiology Score III; APTT, Activated partial thromboplastin time; BMI, Body mass index; BUN, Blood urea nitrogen; CI, Confidence interval; COPD, Chronic obstructive pulmonary disease; OASIS, Oxford Acute Severity of Illness Score; OR, Odds ratio; PT, Prothrombin time; SA-AKI, Sepsis associated acute kidney injury; SOFA, Sequential Organ Failure Assessment; RR, Respiratory rate; WBC, White blood cell.

Table S3. Multivariate logistic regression analysis of sex for severe SA-AKI

| Model | OR (95%CI) | *p-*value |
| --- | --- | --- |
| Model 1 | 1.142(0.981-1.328) | 0.086 |
| Model 2 | 1.209(1.019-1.434) | 0.03 |
| Model 3 | 1.147(0.962-1.368) | 0.127 |
| Model 4 | 1.160(0.904-1.488) | 0.243 |
| Model 5 | 1.175(0.912-1.513) | 0.211 |

**Notes:** Adjusted covariates: Model 1= sex (women=referent).

Model 2= Model 1 + age, BMI, ethnicity and admission type.

Model 3=Model 2 + comorbidities (hypertension, coronary atherosclerosis, heart failure, diabetes mellitus, COPD, cerebral infarction, chronic liver disease, chronic kidney disease, tumor).

Model 4=Model 3 + other variables with *p*<0.1 in the univariate analysis (APS III, OASIS, SOFA, MAP, temperature, WBC, platelet, PH, bicarbonate, BUN, creatinine, potassium, sodium, chloride, glucose, PT, APTT, lactate, vasopressor use, mechanical ventilation use, diuretic, aminoglycoside, statin, ACEI/ARBs).

Model 5 was adjusted for all baseline variables.

**Abbreviations:** ACEI/ARBs, Angiotensin-converting enzyme inhibitors/angiotensin receptor blockers; APS III, Acute Physiology Score III; APTT, Activated partial thromboplastin time; BMI, Body mass index; BUN, Blood urea nitrogen; CI, Confidence interval; COPD, Chronic obstructive pulmonary disease; OASIS, Oxford Acute Severity of Illness Score; OR, Odds ratio; PT, Prothrombin time; SA-AKI, Sepsis associated acute kidney injury; SOFA, Sequential Organ Failure Assessment; RR, Respiratory rate; WBC, White blood cell.

Table S4. Analysis for the effect of comorbidities on the incidence of SA-AKI

| Variables | n | OR (95%CI) | *p*-value | |
| --- | --- | --- | --- | --- |
| Hypertension | |  | |  |
| NO | 3792 | 1.028(0.810-1.306) | | 0.819 |
| YES | 2671 | 1.300(0.977-1.730) | | 0.072 |
| Coronary atherosclerosis | | | |  |
| NO | 5365 | 1.155(0.952-1.402) | | 0.144 |
| YES | 1098 | 1.060(0.615-1.828) | | 0.833 |
| Heart failure | |  | |  |
| NO | 4415 | 1.094(0.889-1.346) | | 0.396 |
| YES | 2048 | 1.209(0.811-1.801) | | 0.352 |
| Diabetes mellitus | |  | |  |
| NO | 4755 | 1.107(0.904-1.356) | | 0.325 |
| YES | 1708 | 1.232(0.809-1.876) | | 0.330 |
| COPD |  |  | |  |
| NO | 5902 | 1.171(0.971-1.413) | | 0.099 |
| YES | 561 | 0.467(0.188-1.165) | | 0.103 |
| Cerebral infarction | | | |  |
| NO | 5790 | 1.072(0.886-1.298) | | 0.476 |
| YES | 673 | 2.062(1.088-3.905) | | 0.026 |
| Chronic liver disease | | | |  |
| NO | 6365 | 0.139(0.950-1.365) | | 0.16 |
| YES | 98 | 0.106(-) | | 1.000 |
| Chronic kidney disease | | | |  |
| NO | 5051 | 1.162(0.955-1.414) | | 0.133 |
| YES | 1412 | 1.089(0.663-1.790) | | 0.735 |
| Tumor |  |  | |  |
| NO | 5354 | 1.205(0.984-1.476) | | 0.071 |
| YES | 1109 | 0.917(0.597-1.410) | | 0.694 |

**Notes:** Multivariate logistic regression analysis of sex for AKI. Model was adjusted for all baseline variables, including age, sex, BMI, ethnicity, admission type, hypertension, coronary atherosclerosis, heart failure, diabetes mellitus, COPD, cerebral infarction, chronic liver disease, chronic kidney disease, tumor, APS III, OASIS, SOFA, heart rate, MAP, RR, temperature, SpO2, WBC, hemoglobin, platelet, PH, bicarbonate, BUN, creatinine, potassium, sodium, chloride, glucose, PT, APTT, lactate, vasopressor use, mechanical ventilation use, diuretic, aminoglycoside, statin, ACEI/ARBs.

Figure S1. A graph showing the covariate balance of the matching balance effect.


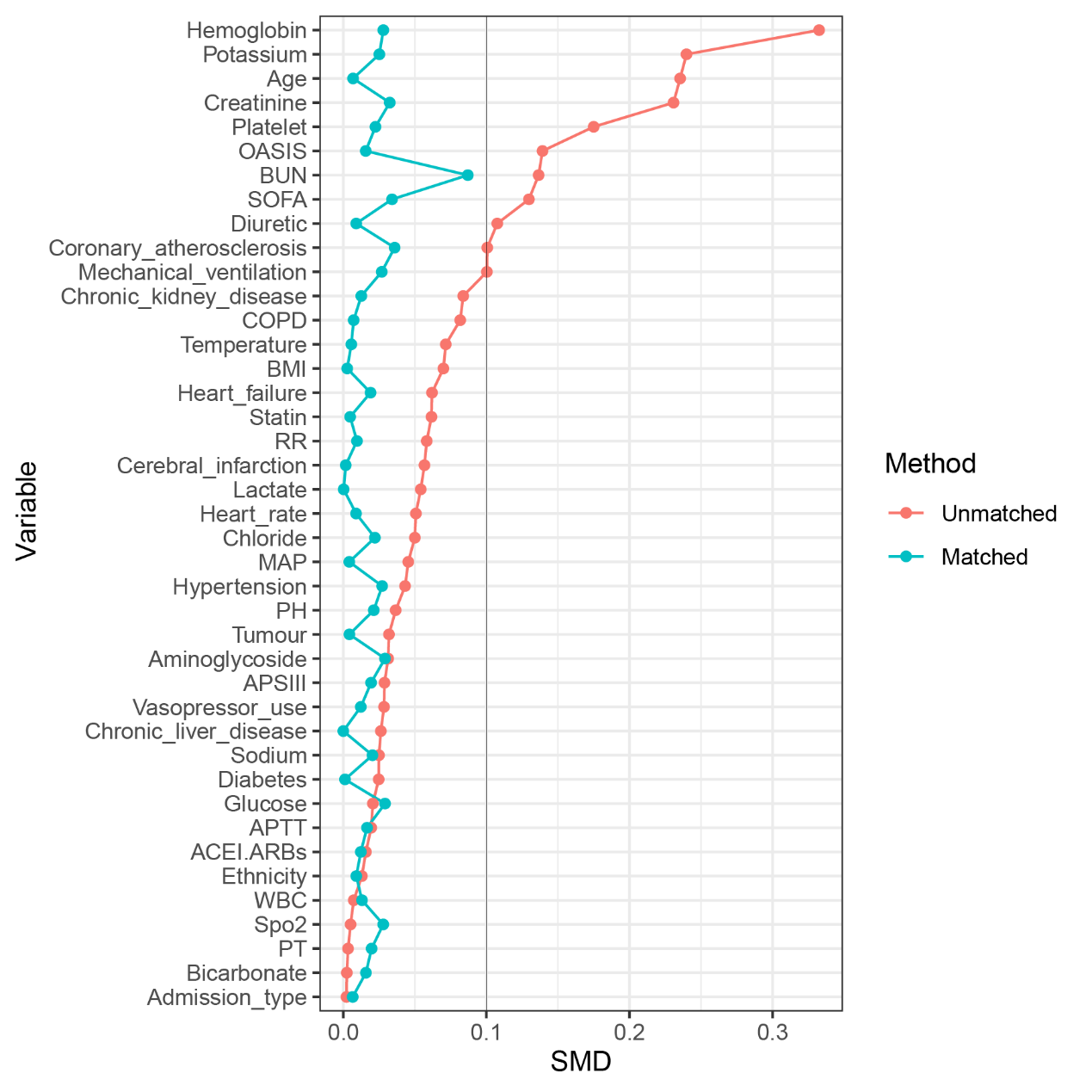

Supplement: Supplementary file 1 [file DataSheet_1.docx]
